# Annotations

**Published:** 1906-11-10

**Authors:** 


### ANNOTATIONS.

### Schools and Fevers.

There seems to be no diminution of the deaths from infectious diseases nor of their incidence. In one week eight deaths from measles, ten from scarlet fever, thirteen from diphtheria, thirteen from whooping-cough, and twelve from enteric fever were recorded from the London district. The admissions in the same week to the London Fever Hospitals of the Metropolitan Asylums Board also showed an increase. Scarlet fever cases numbered 533, diphtheria, 135, and enteric fever 31. In the Hampton district the Sanitary Committee have reported that they had instructed their inspector of nuisances to serve warning notices upon the parents of children suffering from measles who send other children living in the same house to school. The Medical Officer of Health for the Hitchin Urban District Council stated in his report that the evidence was almost conclusive that schools were the chief agent in the dissemination of infectious diseases, and advised the exercise of the power of closure. The Newton Council, referring to the insanitary condition of the Penygloddfa non-provided school, proposed to take action against the managers of schools which are what one of the Councillors called, "poison dens." It was decided by this Council to prosecute the managers for permitting the place to be in such a scandalous condition and thus endangering the health of the 180 children attending there. There seems to be an attempt among medical officers to devolve the duty of reporting and preventing such infectious cases upon the parents. It seems to us that the better method is that adopted by the Newton Council. Notices should be served not on the parents but on the headmasters, school-managers, and the medical officers of health of these disease-breeding "dens" for neglecting the most elementary precautions in safeguarding the health of the children who are compelled to attend them. A precaution recognised by all the leading authorities to be absolutely necessary in checking school outbreaks is that of sterilising the schoolroom floors nightly with an efficient disinfectant. The parent knows nothing about sanitation, and naturally looks to the medical officer of the school and to the sanitary authorities to take the lead in such matters. This applies with tenfold force to the schools in densely populated areas and in great towns.

### The Dangers of "Dry-Cleaning."

Till recently the cleaning of garments that would not stand ordinary washing was left to regular firms, who kept their methods secret, and had suitable premises for the work. Even then accidents were not unknown, but they were infrequent. Now, however, with the spread of shallow scientific knowledge many people have learned what are the fundamental ingredients of "dry-cleaning," and the information that you may clean your gloves with naphtha and your gowns with petrol is passed around from one woman to another, till *nettoyage à sec* has become a regular function of the suburban housekeeper. Unfortunately, the information rarely includes advice as to the precautions necessary in using such inflammable materials. Recently a dressmaker was summoned before the magistrate at Westminster for keeping a quantity of petroleum without a license. The intention of the prosecution was not so much to have the offender severely punished as to make known the risks attendant on meddling with petrol. Indeed, the poor creature had already been punished severely enough. She had bought a two-gallon tin of petrol for the purpose of cleaning some skirts and a carpet. She had done the work in her kitchen, and had applied a light to the gas. The atmosphere of the apartment must have been impregnated with the spirit she had been using, for the result was an explosion and a fire, by which she herself was badly injured. As she had committed a legal offence by keeping the petrol on her premises—though doubtless with as little consciousness of wrong as of danger—she was brought before the magistrate. It is very desirable that the danger attending the use of petrol should be better known. The number of fires due to petroleum spirit has doubled during the last year, and even chauffeurs, who ought to know the risks of the material with which they work, have caused fires by bringing artificial lights into contact with places where there was a leakage of petrol. The dry-cleaning enthusiast has also brought about accidents by pouring waste spirit into drains. Considering how little a London house is adapted for such operations, and the mischief that may follow an accident, the housewife would be well advised to leave the task and the petrol required for it is severely alone.

### The Municipal Treatment of Trachoma.

Under this title the editor of the *Ophthalmoscope* draws attention to a somewhat curious position which has arisen in connection with the presence of children suffering from trachoma in the Poor-law schools of London. The Metropolitan Asylums Board, it seems, has provided two special schools for children with ophthalmic diseases—the one at Brentwood, the other at Swanley—and has provided the necessary staff and equipment. But the difficulty is to secure inmates for these institutions, and to such a pass have matters come that the Local Government Board has issued a circular letter to the various Boards of Guardians calling attention to the facilities provided. There is, however, no existing provision for the detection of cases of trachoma in the Poor-law schools, nor can the Guardians be compelled to transfer any child from their own jurisdiction to the care of the special schools at Brentwood and Swanley. What is wanted is a systematic inspection of all the children in Poor-law schools, and power to compel the Guardians to remove any child who is properly certified to the special ophthalmic schools. Even on the ground of expense it is a serious matter to allow children to lose their eyesight, and this more particularly when, by early and efficient treatment, such a disaster could have been prevented. It must also be remembered that trachoma has dangerous possibilities of epidemic extension, and that a "penny-wise" policy in dealing with the individual may sooner or later prove "pound-foolish" in reference not only to the one primarily concerned, but also to many others.

